# 181. RSV Vaccine Effectiveness Against RSV-Associated Hospitalization Among US Adults Aged 60 Years and Older Over Two RSV Seasons

**DOI:** 10.1093/ofid/ofaf695.060

**Published:** 2026-01-11

**Authors:** Diya Surie, Wesley H Self, Katharine A Yuengling, Yuwei Zhu, Cassandra A Johnson, Paul W Blair, Kevin Ma, Carlos G Grijalva, Fatimah S Dawood

**Affiliations:** Centers for Disease Control and Prevention, Atlanta, Georgia; Vanderbilt University Medical Center, Nashville, Tennessee; Centers for Disease Control and Prevention, Atlanta, Georgia; VUMC, NASHVILLE, Tennessee; Vanderbilt University Medical Center, Nashville, Tennessee; Division of Infectious Diseases, Vanderbilt University Medical Center, Nashville, Tennessee; Centers for Disease Control and Prevention, Atlanta, Georgia; Vanderbilt University Medical Center, Nashville, Tennessee; CDC, Atlanta, Georgia

## Abstract

**Background:**

A single dose of RSV vaccine has been recommended for certain adults aged ≥60 years since 2023 and was effective against hospitalization during the first season; however, duration of protection is unknown. We evaluated RSV vaccine effectiveness (VE) against RSV-associated hospitalization among adults aged ≥60 years over two RSV seasons (2023–2024 and 2024–2025).

**Methods:**

Adults aged ≥60 years were included in this test-negative, case-control analysis if they were hospitalized with acute respiratory illness at any of 26 hospitals in 20 US states during October 1–March 31 of each season and had respiratory virus testing within 10 days of illness onset. Nasal swabs were obtained and tested by reverse transcription-polymerase chain reaction for RSV, SARS-CoV-2, influenza, and hMPV viruses. Case-patients tested positive for RSV only and control-patients tested negative for RSV, SARS-CoV-2 and influenza. Demographic and clinical data were obtained through patient interview and electronic medical record (EMR) review. RSV vaccination status was determined from vaccine registries, EMR, and plausible self-report. RSV vaccination was defined as RSV vaccine receipt ≥14 days before illness onset. Multivariable logistic regression compared the odds of RSV vaccination between case- and control-patients. VE was calculated as (1 – adjusted odds ratio) x 100%. Models were adjusted for age, sex, race and ethnicity, geographic region, and calendar month. Analyses were stratified by RSV vaccine receipt in the same or prior season relative to illness onset.

**Results:**

Of 6,271 adults aged ≥60 years, 771 (12.3%) were RSV case-patients and 5,500 (87.7%) were control-patients. Median (IQR) age was 72 (66–80) years and 1617 (25.8%) were immunocompromised. A total of 58/771 (7.5%) case-patients and 816/5500 (14.8%) control-patients were vaccinated. Among adults aged ≥60 years, VE against RSV-associated hospitalization over two seasons was 57% (95% CI, 43%–68%) with a VE of 72% (95% CI, 54%–83%) for those vaccinated in the same season before illness onset and 44% (95% CI, 20%–60%) for those vaccinated in the prior season.

**Conclusion:**

RSV vaccines were effective against RSV-associated hospitalization over two seasons. Ongoing monitoring is needed to determine the optimal RSV revaccination interval.

**Disclosures:**

All Authors: No reported disclosures

